# Vertical and horizontal bone loss following alveolar socket preservation using bone grafts and autologous platelet concentrates vs bone grafts alone: a systematic review and meta-analysis

**DOI:** 10.1038/s41405-025-00306-y

**Published:** 2025-02-06

**Authors:** Shivang Davda, Rawand Shado, Ines Novo Pereira, David Madruga, Haidar Hassan

**Affiliations:** 1 Rey Juan Carlos University Av. de Atenas, S/N 28922 Alcorcón, Madrid Spain; 2 Barts & The London School of Medicine & Dentistry, Queen Mary University, Institute of Dentistry, Royal London Dental Hospital, Turner Street, London, E1 2AD United Kingdom; 3 Faculdade de Medicina Dentária, Universidade do Porto, Rua Dr. Manuel Pereira da Silva, 4200-393 Porto, Portugal; 4 Barts & The London School of Medicine & Dentistry, Queen Mary University, Centre for Cutaneous Research, Blizard Institute of Cell and Molecular Science, 4 Newark Street, Whitechapel, London, E1 2AT United Kingdom

**Keywords:** Oral surgery, Dental conditions

## Abstract

**Background:**

Socket preservation is a proactive approach that limits bone loss after tooth extraction to maintain adequate bone volume, height and width. Many methods have proven effective in achieving socket preservation, including using various bone grafts and autologous platelet concentrates (APCs). Combining these two methods may lead to improved results in socket preservation and patient outcomes.

**Aims:**

To compare the combined use of APCs and bone grafts in socket preservation, with the use of bone grafts alone. Primary outcomes were radiographic vertical bone loss (VBL) and horizontal bone loss (HBL).

**Methods:**

A search on Pubmed, Scopus, Embase and Google Scholar databases was conducted to identify human studies using APCs in extraction sockets between January 2014 and August 2024. The inclusion criteria involved comparative human studies ranging from evidence levels II to III (Oxford Centre for Evidence-Based Medicine Levels of Evidence). For assessing bias in the included studies, the Cochrane Risk of Bias tools were used. The Grading of Recommendations Assessment, Development, and Evaluation (GRADE) approach was used to determine the quality of evidence available.

**Results:**

A total of five randomised controlled trials (RCTs) were included in the analysis. Studies included the use of platelet rich fibrin (PRF), injectable platelet rich fibrin (i-PRF), advanced platelet rich fibrin (A-PRF), advanced platelet rich fibrin plus (A-PRF+) and concentrated growth factors (CGF). The risk of bias was judged high and moderate for two out of five RCTs. The analysis revealed a combined effect size for VBL reduction, with a standardized mean difference (SMD) of −0.83 (*p* < 0.001; 95% confidence interval (CI) = [−1.2, −0.57]; I² = 73.13%). For HBL reduction, the combined effect size was SMD = −0.72 (*p* < 0.001; 95% CI = [−1.08, −0.37]; I² = 68.34%). The overall evidence quality rating for the use of APCs in combination with bone grafts to reduce VBL during socket preservation was assessed as moderate, whereas to reduce HBL it was determined to be low.

**Conclusion:**

The current literature demonstrates the added benefits of APCs combined with bone grafts in alveolar socket preservation compared to bone grafts alone in reducing vertical and horizontal bone loss. However, based on the GRADE assessment, the quality of evidence was judged low-to-moderate. Further randomised clinical studies would increase the certainty of the evidence.

## Introduction

It has been reported that within the first year following extraction, the average resorption of bone width can reach up to 50%, accounting for a loss of 5–7 mm. Furthermore, approximately 66% of this resorption occurs within the initial three months [1]. To address this issue, alveolar socket preservation techniques have been developed. These techniques involve filling completely contained extraction sockets with materials to reduce dimensional changes. While some review articles use the terms, socket and ridge preservation, interchangeably, alveolar ridge preservation is defined as any procedure designed to mitigate the negative effects of post-extraction resorption, maintain the soft and hard tissue contour of the ridge, promote bone formation within the socket and facilitate implant placement in a prosthetically driven position [2].

Essentially, both socket preservation and alveolar ridge preservation aim to minimise the resorptive process following tooth extraction. Studies have shown that implementing socket preservation techniques can enhance prosthodontic and aesthetic outcomes when implants are used [3]. In fact, the need for hard tissue augmentation at the time of implant placement was found to be five times higher when socket or ridge preservation was not performed on the day of extraction [4, 5].

Various methods have been employed for socket preservation, ranging from simple techniques such as the use of membranes and bone grafts to more advanced approaches involving autologous platelet concentrates (APCs) or combinations of APCs with bone grafts. For over 20 years, APCs have been applied in dentistry in various contexts, including the regeneration of bone, periodontium, and the pulp-dentine complex [6, 7]. These concentrates contain a high concentration of growth factors that promote wound healing and stimulate tissue regeneration [8].

Research indicates that socket preservation with APCs is superior to no preservation at all [9] and enhances soft tissue healing. Furthermore, combining APCs with bone substitutes for socket preservation may lead to improved outcomes, including increased bone quality and enhanced osteogenesis.

## Autologous platelet concentrates mechanism in regeneration

Tissue repair and regeneration are regulated by a network of cellular components and molecular signals, with platelets playing a role in these processes. Platelets release growth factors that promote tissue repair and regeneration [10, 11]. These growth factors are transported within a fibrin matrix, which forms a mesh-like scaffold that acts as a carrier for these aggregates, transforming a clot into a firm and stable haemostatic plug [7, 9]. Fibronectin also contributes to these processes by promoting neovascularisation [12]. Among the various growth factors involved, vascular endothelial growth factor (VEGF) is significant for its roles in angiogenesis and vascular permeability [7, 13]. Additionally, platelet-derived growth factors (PDGF) initiate bone regeneration [14]), while transforming growth factor-beta (TGF-β) controls the rate of bone formation [15].

All APCs involve the collection of a patient’s blood, which is subsequently processed through a centrifuge. However, there is currently no standardised protocol for any specific type of platelet aggregate. Variations can occur in terms of centrifugal force, speed, duration, temperature, vial size, anticoagulant used, materials comprising the vial and the surface roughness of the material. These differences in protocols result in a multitude of aggregate concentrations. These variations have evolved to produce three generations of APC (Table 1). The first generation includes platelet rich plasma (PRP), leukocyte platelet rich plasma (L-PRP) and platelet rich in growth factors (PRGF). The second generation comprises platelet rich fibrin (PRF), leukocyte platelet rich fibrin (L-PRF), injectable platelet rich fibrin (i-PRF), advanced platelet rich fibrin (A-PRF) and advanced platelet rich fibrin plus (A-PRF+). The third generation introduces concentrated growth factors (CGF). However, Alrayyes (2022) [16] highlights the lack of standardised preparation protocols for specific APC formulation.

**Table 1:** Evolution of Autologous Platelet Concentrates (APCs).

First generation	
PRP	• Introduced in the 1980s (Sun et al., 2022) • Involves collecting a patient’s blood in a vial containing an anticoagulant, followed by two centrifugation stages. • Depending on the conditions of the second centrifugation stage, the resulting PRP can be classified as either leukocyte-rich platelet plasma (L-PRP) or pure platelet-rich plasma (P-PRP), with the primary distinction being the leukocyte content. • There is currently no standardized protocol for either centrifugation stage; there are approximately 40 different protocols for obtaining PRP (Pavlovic et al., 2016).
PRGF	• Introduced in 1999 by Eduardo Anitua. • Uses sodium citrate as the anticoagulant and calcium chloride as an activator (similar to PRP). • Employs a one-stage process involving only a single centrifugation.
Second generation	
PRF	• Developed by Joseph Choukroun in 2001. • Characterized by the absence of anticoagulants or additives. • Utilizes only one centrifugation stage. • Upon blood collection, the sample must be quickly placed in the centrifuge and spun to prevent premature coagulation. In this process, fibrinogen concentrates at the top of the tube, and when mixed with circulating thrombin, it transforms into fibrin, trapping platelets in the process. A fibrin clot forms in the middle of the tube, with red blood cells settled at the bottom and plasma above (Dohan et al., 2006).
A-PRF	• Subsequent development introduced by Ghanaati et al. in 2014. • Involves spinning larger tubes (10 ml compared to the 9 ml used for PRF) at a lower rotational speed (1500 rpm) for an extended duration (14 minutes). • The adjustment in centrifugation parameters facilitates the release of more leukocytes and greater quantities of growth factors such as TGF-β1, PDGF, and VEGF (Fujioka-Kobayashi et al., 2017).
A-PRF+	• Iteration of A-PRF that further reduces the centrifugal speed and processing time, resulting in increased growth factor release (Miron & Choukroun, 2017).
Third generation	
CGF	• Introduced by Sacco in 2005. • Also uses a single centrifuge (Chen and Jiang, 2020). • Produces a denser fibrin matrix by employing a variable centrifugation speed, alternating between acceleration and deceleration (unlike PRF, which maintains a constant speed throughout the process). This variation enhances the conversion rate of fibrinogen to fibrin. • The properties of the resulting CGF can differ based on the materials used for the tubes; rough inner walls yield a dense gel, while smooth walls produce a looser gel. • If anticoagulants are added prior to centrifugation, a liquid CGF is obtained (Sun et al., 2022). • A comprehensive review by Chen and Jiang (2020) found no evidence that CGF is superior to previous generations of platelet-rich aggregates, indicating a need for further studies to assess any potential benefits of using CGF.

A detailed comparison of the different generations of APCs, highlighting the key differences in preparation protocols, centrifugation methods, and clinical applications.

### Bone and bone substitutes

Several types of bone substitutes, or grafts, have been reported, including autogenous, allogenous, xenogenous, alloplastic (synthetic) and phytogenic. Table 2 [17–19] summarises the differences between the distinct types of bone grafts.

**Table 2:** Comparison of the relevant bone substitutes.

Type	Source	Examples	Advantages	Disadvantages
Autograft	Same patient	Cortical Cancellous	• Osteoinduction • Osteogenic • Osteoconductive • High safety – no risk of transmission	Limited availability and quantity Delayed incorporation Second surgery may be required Surgery time is increased Morbidity is increased Additional cost Increased bleeding Unpredictable resorption
Allograft	Same Species	FDBA DFDBA	• Osteoconduction • Osteoinduction • No donor site morbidity • Less pain • High availability	Rejection of graft Delayed incorporation Inadequate vascularisation Risk of disease transmission Ethical concerns
Xenograft	Another species	Anorganic Bovine Bone	• Osteoconduction • Osteoinduction • More economic • High availability • Less pain	Risk of disease transmission Lacking in osteoconductive properties As granular structure, can be harder to hold in surgical sites Not fully resorbable (Valentini and Bosshardt, 2018)
Alloplastic (synthetic) graft	Lab-made	β-Tricalcium phosphate	• Osteoconduction • Biocompatability • Less pain • Unlimited supply • Easy sterilisation • Easy storage • Injectbility	Brittle Low compression and tensile strength
		Hydroxyapatite	• Unlimited quantities • Biocompatibility	Delayed resorption rate Low mechanical strength Lacks the microporous structure of bovine hydroxyapatite
		Calcium phosphate cement	• Osteoconduction • Self-setting • Injectability • Gradual absorption and replacement by new bone • Mouldability • Biocompatability	Brittle Low speed of cell adhesion Low strength, not useful in load bearing areas
		Calcium sulphate	• Low cost • Readily available • High mouldability • Biocompatability • Short setting time	Limited osteoconductivity due to not being porous Rapid resorption
Phytogenic	Red Algae	Algae-based	• Osteoconduction • Good resorbability • Low immunogenicity	Lack of studies
	Marine Coral	Coral-based	• Osteoconduction • Good compressive strength • Improved cell adhesion • Low immunogenicity	Brittleness Poor resorption Low tensile strength

A comparison of different bone substitutes, categorising them by their source and highlighting their key advantages and disadvantages.

### Rational behind this review

Given that both bone grafts and APCs have independently demonstrated benefits in socket preservation compared to spontaneous healing, it is worthwhile to explore whether the combination of these two materials might yield additional advantages. Therefore, this review aims to investigate whether the combination of APCs and bone grafts results in reduced vertical bone loss (VBL) and horizontal bone loss (HBL) compared to the use of bone grafts alone.

## Methods

The Preferred Reporting Items for Systematic reviews and Meta-Analyses (PRISMA) [20](Supplement 1) [21] was followed for reporting this review. The PICO framework [22] was modified to include (T) for time and used to structure the reporting of eligibility criteria:

(P) Population: Adult humans requiring tooth extraction

(I) Intervention: APC + bone graft for socket preservation postextraction

(C) Comparison: : bone graft only for socket preservation postextraction

(O) Outcomes: radiographic vertical and horizontal bone loss

(T) Time: 4–6 months

### Search strategy

PubMed, Scopus, Embase and Google Scholar were the main databases used for conducting the search for articles in this review. Supplement 1a presents the key search terms used for articles retrieval. The databases were last searched on August 24^th^, 2024.

### Study selection

The eligibility criteria (Supplement 1b) was for randomised controlled trials (RCTs) only and ensured that the selected studies focused on comparing the dual approach (APCs and bone grafts) against solely bone grafts in reducing vertical and horizontal bone loss as a socket preservation technique.

### Inclusion criteria

We included studies conducted in hospital or clinical settings with an adult population aged 18–75 years and restricted to RCTs. Eligible studies needed to use APCs in extraction sockets and report radiographic outcomes related to vertical and horizontal bone loss. Studies comparing APCs combined with bone grafts versus bone grafts alone were included. The investigation period was restricted to studies conducted between January 1, 2014 - August 24, 2024, with no restrictions on the type of APCs, sample size or geographic location.

### Exclusion criteria

Studies were excluded if they lacked a graft-only group, did not report radiographic bone loss, involved paediatric or special needs populations. We excluded studies focused on restorative or endodontic treatments, non-dental procedures, animal studies or non-research publications such as reviews, editorials and conference abstracts. Duplicate studies and those not published in English were also excluded.

Study selection was conducted by two independent reviewers (SD, RS) in the following stages: 1) Initial screening of potentially suitable titles and abstracts against the inclusion criteria to identify potentially relevant papers. 2) Screening of the full papers identified as possibly relevant in the initial screening. 3) Studies were excluded if not meeting the inclusion criteria. Following the screening of titles and abstracts, the studies included by both reviewers were compared. In case of disagreement, consensus on which studies to include was reached by discussion. When necessary, a third reviewer made the final judgement (HH).

### Data collection

Data collection for this systematic review was conducted independently by two reviewers (SD, RS). Any discrepancies between the reviewers were resolved by a third author (HH), who conducted an additional review of the extracted data.

For each included study, information was gathered regarding the authorship, year of publication and country of origin. Key demographic variables were extracted, including the sites of extraction, the sex and age of the participants. The methodology of each study was assessed, focusing on the use of APCs and the protocols employed for their preparation, investigated sites, postoperative bone measurement time (months) and bone level results. The studies were then categorised according to the Oxford Centre for Evidence-Based Medicine Levels of Evidence (OCEBM) classification system [23].

### Data preparation

For continuous variables, such as age, when distinct values were provided for each respective group and no statistically significant difference was observed between the groups, we combined the mean and standard deviation values into a single entry in our data tables. This ensured comparability of the reported parameters. In instances where a specific data point was entirely absent, we systematically documented as “Not Reported” (NR) in our analysis.

### Risk of bias

The Risk of Bias 2 (RoB2) assessment tool [24] was used to assess the risk of bias for RCTs (Level II). The bias category of ‘Some concerns’ was labelled as ‘Moderate’ for the purpose of font size clarity in the generated tables.

For each study, the overall bias was given based on the highest bias score for each decision category. For example, if the highest score of ‘high’ was estimated for one or more decision categories, then the overall bias was considered ‘high’.

### Data analysis

The Grading of Recommendations Assessment, Development, and Evaluation (GRADE) approach [25] was used to determine the certainty for a body of evidence available in the identified studies for this evidence-based review. Each component of the GRADE approach was independently assessed by three reviewers (SD, RS, HH). In cases of disagreement, another author (INP) facilitated a discussion to ensure consensus was reached.

In this meta-analysis, the standardized mean difference (SMD) was calculated using Hedge’s d. It was anticipated that various studies might use different APCs. To address this potential heterogeneity, a random-effects model was employed to mitigate its effects. When two or more studies used the same APC, a subgroup analysis was performed to assess the specific effects associated with that particular APC.

### GRADE

To assess the indirectness of the evidence, we analysed the differences in the types of APCs used and the characteristics of patient populations. For inconsistencies, the statistical I² test and the Cochran’s Q test were employed to evaluate heterogeneity, alongside visual inspection of the confidence intervals. The I² heterogeneity thresholds were based on the Higgins and Thompson classification [26], categorising heterogeneity as low (25%), medium (50%) and high (75%).

For imprecision, the analysis focused on the overlap of confidence intervals with the no-effect threshold (SMD = 0) and the effect size thresholds defined by Cohen’s classification, specifically small (0.2), moderate (0.5), and large (0.8). A precise effect is indicated when the 95% confidence interval extends beyond the moderate (0.5) effect threshold.

To evaluate publication bias, visual inspection of the funnel plot for asymmetry was conducted, along with the application of Egger’s Test.

## Results

### Studies included

**Fig. 1:**
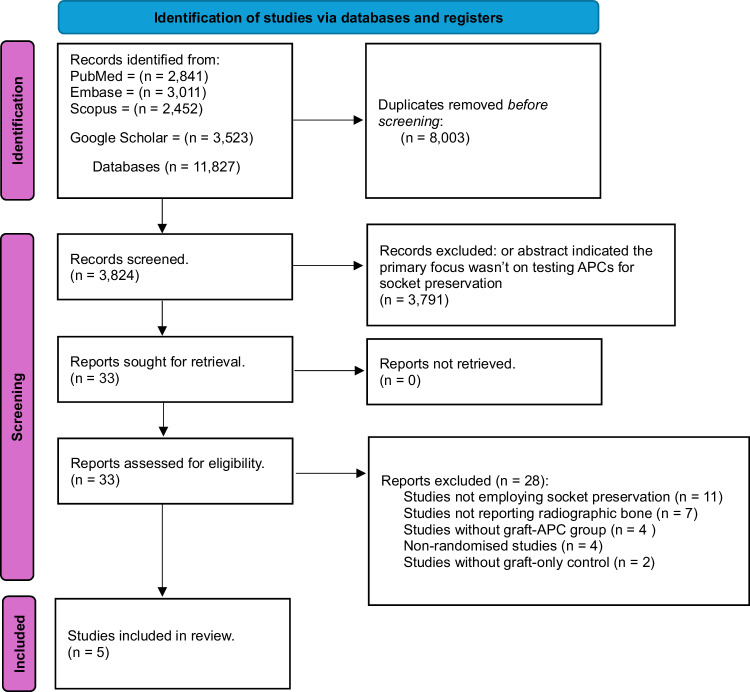
PRISMA flowchart. A flowchart demonstrating the identification, screening and the inclusion process of the included articles in this review.

Figure 1 shows the PRISMA flowchart [21] representing study selection and inclusion. The initial search resulted in 11,827 papers for all databases combined. This was trimmed down to 3824 after duplicates were removed. Following the first-stage screening of titles and abstracts, 33 articles (considered potentially suitable by at least one reviewer) qualified for full-text screening. After full-text reading; five studies, with 126 extraction sites in total and 4–6 months follow up, met the inclusion criteria, and 28 papers were excluded.

### Study characteristics

Table 3 [20, 27–30] reports the studies and their characteristics, 5 RCTs. The table shows the authors, year of publication, country of publication, study type, level of evidence, risk of bias, groups characteristics, APC preparation protocol, investigated sites, postoperative bone measurement time (months) and bone level results. Studies were classified as level of evidence II for RCTs.

**Table 3:** Summary of clinical studies comparing autologous platelet concentrates and bone substitutes for bone regeneration.

First author, year, country, study type - level of evidence (risk of bias)	Outcome measures	Population characteristics	Test vs Control Groups	APC preparation protocol	Sites	Postoperative bone measurement timing (months)	VBL in control group	VBL in test group	HBL in control group	HBL in test group
Abaza, 2023, Egypt RCT - II (Low)	CBCT radiographic bone level soft tissue thickness and clinical bone width histological assessment and histomorphometric analysis of core bone biopsies	Total patients (n): 24 Females (n): 13 Males (n): 11 Age (years): 31.60 ± 5.05 (20–50)	Test: DBBM (cerabone®) + **i-PRF [liquid]** Sites: 12 **vs** Control: DBBM (cerabone®) Sites: 12	iPRF • 10 mL venous blood samples were collected from the patient • No anticoagulants. • Centrifuged in a plain plastic glass-coated tube. • 700 rpm x 3 min	maxillary non-molars	4	0.98 ± 0.18	0.53 ± 0.11	1.27 ± 0.9	0.63 ± 0.9
Keranmu, 2022, China RCT - II (High)	CBCT radiographic bone level changes of alveolar bone contour postoperative pain & healing	Total patients (n): 38 Females (n): 23 Males (n): 15 Age (years): 28.89 ± 2.7	Test: DBBM (cerabone®) + **CGF [membrane]**+ CM (Bio-Oss®) Sites: 19 **vs** Control: DBBM (cerabone®) + CM (Bio-Oss®) Sites: 19	CGF • 4 mL Venous blood sample were taken • Samples were collected into sterile vacuum tubes • No anticoagulant • Centrifuge (Medifuge, Silfradent, Italy) • CGF isolated	non-molars	6	2.33 ± 1.902	0.64 ± 1.775	3.15 ± 1.026	1.31 ± 0.930
Yewale, 2021, India RCT - II (Low)	CBCT radiographic bone level Gain in socket fill postoperative pain & swelling	Total patients (n): 20 Females (n): 11 Males (n): 9 Age (years): 35.15 (20 - 55)	Test: HA-β TCP (Sybograf plus ™) + **A-PRF-plus [membrane]** + CM (Collasponge ™) Sites: 10 **vs** Control: HA-β TCP (Sybograf plus ™) + CM (Collasponge ™) Sites: 10	A-PRF plus • 10 mL of venous blood samples were collected • No anticoagulant • 1300 rpm (208 g) x 8 mins	maxillary non-molars	6	1.67 ± 2.774	1.48 ± 2.923	1.83 ± 1.786	2.12 ± 1.154
Clark, 2018, USA RCT - II (Low)	Ridge dimensions after extraction and before implant placement micro-CT and histomorphometric analysis of bone core samples	Total patients (n): 20 Females (n): NR Males (n): NR Age (years): 58	Test: FDBA (AlloOss®) + **A-PRF [clot]** + CM (Collaplug®) Sites: 10 **vs** Control: FDBA (AlloOss®)+ CM (Collaplug®) Sites: 10	A-PRF • 10 mL venous blood samples taken • Samples collected into sterile glass vacuum tube • No anticoagulant • 1300 rpm (200 g) x 8 mins	non-molars	4	2.2 ± 1.8	1.0 ± 2.3	2.5 ± 1.1	1.9 ± 1.1
Thakkar, 2016, India RCT - II (Moderate)	CBCT radiographic bone level	Total patients (n): 36 Females (n): 15 Males (n): 21 Age (years): (20–55)	Test: DFDBA (NR) + **PRF [clot]** + CM (NR) Sites: 18 **vs** Control: DFDBA (NR) + CM (NR) Sites: 18	PRF • 10 mL venous blood samples were taken • No anticoagulant • 3000 rpm x 10 min	non-molars	6	1.389 ± 0.393	1.083 ± 0.429	1.361 ± 0.703	1.056 ± 0.338

Comparison of different randomized controlled trials (RCTs) assessing the effectiveness of autologous platelet concentrates (APCs) in combination with bone substitutes for bone regeneration.

*i-PRF* injectable platelet rich fibrin, *A-PRF* advanced platelet rich fibrin, *A-PRF-plus* advanced platelet rich fibrin plus, *CGF* concentrated growth factors, *DBBM* Demineralised bovine bone mineral, *FDBA* freeze-dried bone allograft, *DFDBA* demineralized freeze-dried bone allograft, *HA-β TCP* hydroxyapatite and β-tricalcium phosphate, *CM* collagen membrane, *CBCT* cone beam computed topography, *Micro-CT* micro computed tomography, *rpm* revolutions per minute, *VBL* vertical bone loss, *HBL* horizontal bone loss, *APC* autologous platelet concentrate, *NR* not reported.

bold = Type of APC used in the study.

[] = the consistency of the APC used.

A total of five studies were included in the analysis, representing data from four countries: USA, Egypt, India and China.

All studies focused on non-molar extraction sites. A mix of male and female patients were included in all studies, with some reporting a near-equal distribution of sex, while others had a slight female predominance. The patient populations ranged from younger adults (20 years) to older adults (up to 60 years).

Studies included the use of PRF, i-PRF, A-PRF, A-PRF+ and CGF. APC preparation followed standardised protocols within each trial but with variations in centrifugation speeds and times between the studies.

### Risk of bias

**Fig. 2:**
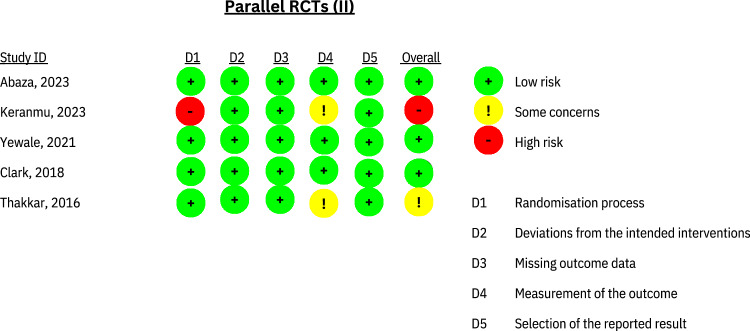
Risk of bias and level of evidence. A diagram showing the bias level within studies.

The risk of bias was categorised as high in one RCT [20], moderate in another RCT [27], and low in three RCTs [28–30]. With regard to the RCT [20] identified as having a high risk of bias, the main concern was the absence of appropriate randomization procedures, which raised questions about the comparability of baseline characteristics between groups (Fig. 2).

### Vertical and horizontal bone loss

In this meta-analysis, vertical and horizontal bone measurements were included based on specific criteria. When multiple surfaces were measured for vertical bone height, only the measurements from the buccal surface were selected for consistency across studies. For horizontal bone measurements, different depths were reported in the included studies; however, only depths ranging from 1 to 3 mm were chosen for this meta-analysis. This ensured that the comparisons were standardised.

**Fig. 3:**
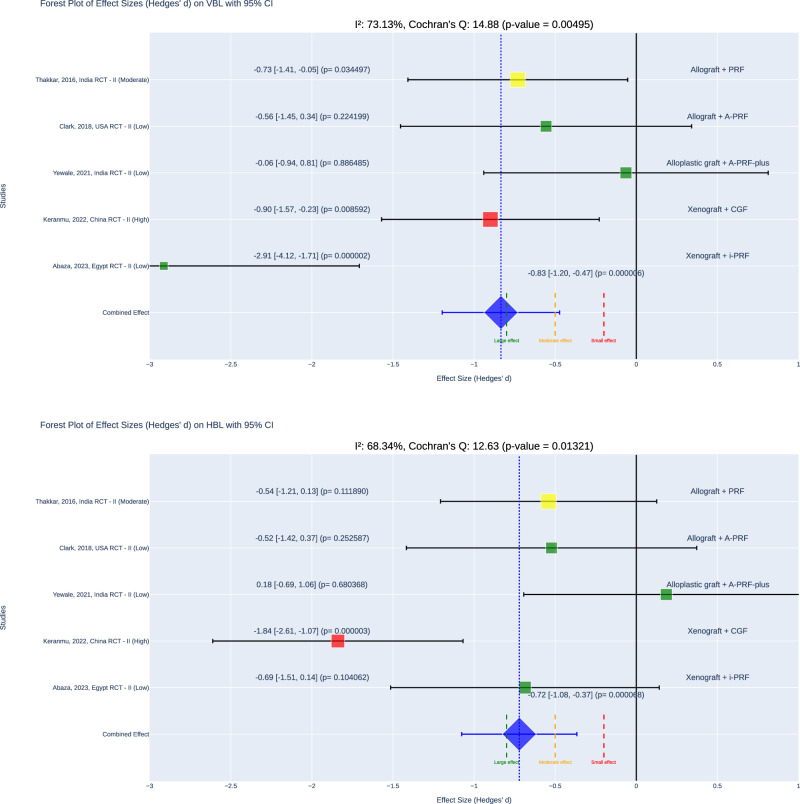
Meta-analysis forest plot. Top plot: A forest plot illustrating effect sizes (Hedges’ d) for VBL with 95% confidence intervals (CIs) across studies. Bottom plot: A forest plot illustrating effect sizes (Hedges’ d) for HBL with 95% CIs across studies.

The meta-analysis revealed a statistically significant reduction in VBL in the APC+ graft groups compared to the graft-only groups, with a combined effect size (Hedge’s d) of −0.83 (*p* < 0.001, 95% confidence interval (CI) = [−1.2, −0.57], I² = 73.13%). The 95% CI indicated that the effect size ranged from medium to large according to Cohen’s classification. For horizontal bone loss (HBL), the APC + graft groups also demonstrated significantly less loss compared to the graft-only groups, with a combined effect size (Hedge’s d) of −0.72 (*p* < 0.001, 95% CI = [−1.08, −0.37], I² = 68.34%). The effect size here varied from very small to large (Fig. 3).

**Fig. 4:**
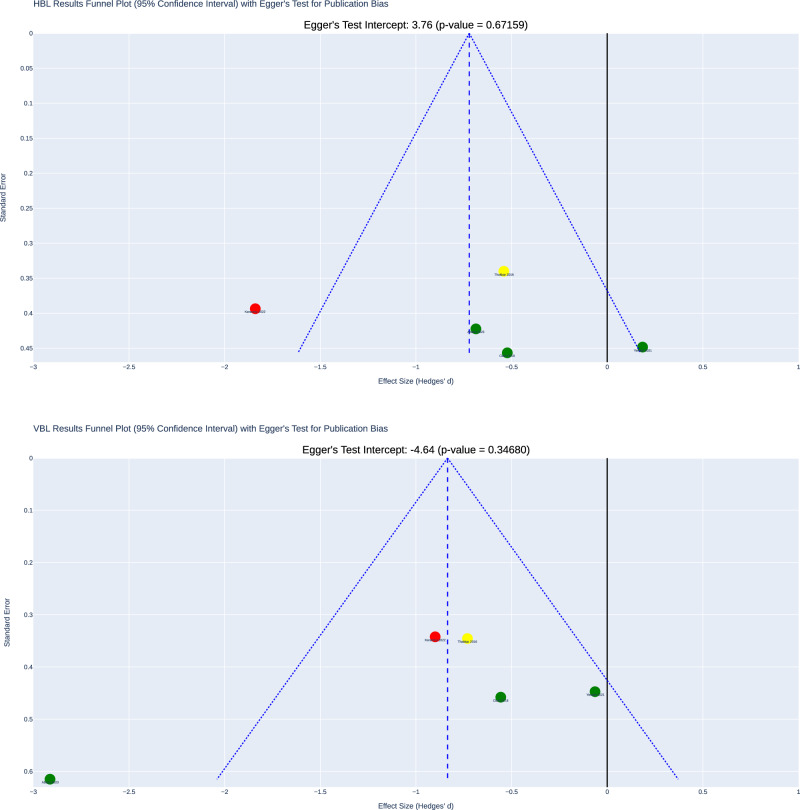
Meta-analysis publication bias. Top plot: Funnel plot assessing publication bias with Egger’s test for VBL with 95% CIs across studies. Bottom plot: Funnel plot assessing publication bias with Egger’s test for HBL with 95% CIs across studies.

The funnel plot (Fig. 4) visually assesses publication bias across the included studies. A symmetrical distribution of studies was observed, suggesting an absence of publication bias. The Egger’s test indicates no statistically significant evidence of publication bias (*p* > 0.05).

### GRADE

The inconsistency in the results for VBL showed high heterogeneity (I² = 73.13%), as well as the results for HBL showed high heterogeneity (I² = 68.34%). Factors contributing to this heterogeneity included differences in surgical techniques, variations in perioperative and postoperative care, the specific type of APC employed, the type of graft utilised, the use of collagen membranes, the site of tooth extraction, and the duration of follow-up across the studies.

All studies performed direct comparisons between bone grafts alone and those combined with an APC; however, the use of different APCs across the studies complicates the reliability of the direct comparisons of their findings.

Publication bias was not detected, as indicated by the symmetry of the funnel plot and Egger’s Test. The 95% CIs for the results extend beyond the no-effect threshold, indicating a range from medium to large effects for reducing VBL [−1.20, −0.47] and from small to medium effects for reducing HBL [−1.08, −0.37]. The 95% CI for VBL suggests a potential for large effect sizes, whereas the 95% CI for HBL indicates effect sizes that are medium at most.

The risk of bias was assessed as moderate to high in two of the five studies. This level of bias undermines the reliability of the results.

There was no evidence to suggest that biases favoured the APC groups within the studies. Given the nominal nature of the APC, observing a dose-response relationship was not applicable.

The overall certainty of the evidence rating for the use of APCs combined with bone grafts in reducing VBL for socket preservation is assessed as moderate, while for reducing HBL is determined to be low (Table 4).

**Table 4:** Summary of evidence quality for APC studies.

	VBL									
	Studies	Risk of Bias	Inconsistency	Indirectness	Imprecision	Publication bias	Is there large effect size?	Is there dose response relationship?	Does confounding biases reduce the effect size?	Overall Quality
Comments	5 RCTs	40% (2/5) of studies have moderate to high risk of bias	High heterogeneity (I² = 71.13%) and one study showing no overlap of the 95% CI. Explained by methodological heterogeneity. No study showed contradicting results to another study.	Direct comparisons between bone grafts alone and those combined with an APC Different APCs used across the studies One study had older patients compared to others (mean age = 58)	95% CI show medium to large effects for reducing VBL [−1.20, −0.47] which is also beyond the no-effect threshold.	Funnel plot and Eager’s test results show no evidence of publication bias.	SMD and 95% CI shows large effect size	Not applicable in the context of APCs	No evidence to suggest biases reduce or increase the effect size.	Moderate ⨁ ⨁ ⨁ ⊝
Score	4	Serious (−1)	Not serious (0)	Serious (−1)	Not serious (0)	Not serious (0)	1	0	0	3

	HBL									
	Studies	Risk of Bias	Inconsistency	Indirectness	Imprecision	Publication bias	Is there large effect size?	Is there dose response relationship?	Does confounding biases reduce the effect size?	Overall Quality
Comments	5 RCTs	40% (2/5) of studies have moderate to high risk of bias	large heterogeneity (I² = 68.34%) Explained by methodological heterogeneity. One study showed largely significant results compared to other studies.	Direct comparisons between bone grafts alone and those combined with an APC Different APCs used across the studies One study had older patients compared to others (mean age = 58)	95% CI show small to large effects for reducing HBL [−1.08, −0.37] which is also beyond the no-effect threshold.	Funnel plot and Eager’s test results show no evidence of publication bias.	95% CI shows large effect size	Not applicable in the context of APCs	No evidence to suggest biases reduce or increase the effect size in favour for a particular APC	Very low ⨁ ⊝ ⊝ ⊝
Score	4	Serious (−1)	Serious (−1)	Serious (−1)	Serious (−1)	Not serious (0)	1	0	0	1

The evidence quality for studies investigating the use of autologous platelet concentrates (APCs), specifically in terms of vertical bone loss (VBL) and horizontal bone loss (HBL).

## Discussion

Currently, no single treatment can entirely prevent changes in the alveolar bone following tooth extraction, and a certain degree of bone loss is deemed inevitable [31, 32]. However, implant surgery conducted after alveolar ridge preservation has been shown to yield higher success rates and improved treatment outcomes compared to immediate implantation following extraction [33].

A series of studies confirmed that various factors can influence alveolar bone resorption, including smoking, different tooth positions, and systemic conditions [34–36]. Autologous platelet concentrates (APCs) have demonstrated clinical benefits, notably in reducing the incidence of alveolar osteitis and postoperative pain [37]

Recent meta-analyses suggested that the use of APCs in socket preservation is advantageous compared to spontaneous healing [38–40]

The purpose of this meta-analysis was to evaluate the effect of combining APC with bone grafts to bone grafts alone in socket preservation.

By standardising the selection of buccal surface measurements for vertical bone height and specifying depths of 1–3 mm for horizontal bone measurements, this analysis ensured consistency and clinical relevance across the included studies.

For VBL, reporting studies demonstrated statistically significant effect sizes for APCs+bone grafts. For instance, Abaza et al. [30] showed an effect size of −2.91 (*p* < 0.001, 95% CI: −4.12, −1.71), indicating a large effect. In contrast, Yewale et al. [29] demonstrated small or negligible effect sizes, suggesting a limited impact within these sites.

**Fig. 5:**
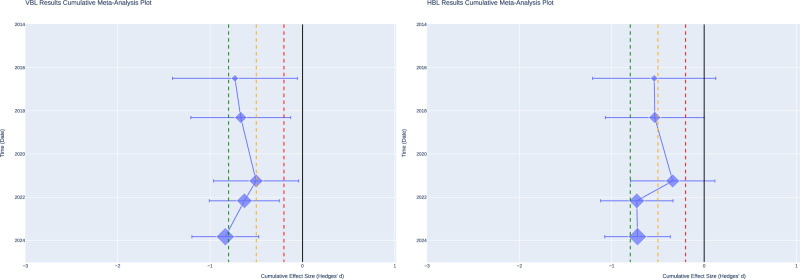
Cumulative meta-analysis plot. Cumulative meta-analysis plot tracking changes in the combined effect size estimates over time for VBL (Left plot) and HBL (Right plot).

The cumulative meta-analysis plot (Fig. 5) illustrates the evolution of effect sizes over time, beginning with Thakkar et al. [27] and extending to Abaza et al. [30]. Early studies indicated larger effect sizes; however, Yewale et al. [29] resulted in a smaller cumulative effect. The cumulative effect size has stabilised at approximately -0.83 in recent years, indicating that the magnitude of the effect has remained consistent as additional data have been collected. These findings suggest that while there is heterogeneity in effect sizes across studies, the overall combined effect reflects a medium-to-large impact, with no evidence of significant publication bias affecting the results.

In the context of horizontal bone loss (HBL), only one study, Keranmu et al. [20], demonstrated a statistically significant effect size for APCs, reporting an effect size of −1.84 (*p* < 0.001, 95% CI: −2.61, −1.07), which indicates a large effect. The remaining studies reported statistically non-significant effects. The cumulative meta-analysis plot (Fig. 5) reveals inconsistencies in the evolution of the effect size, further indicating substantial heterogeneity in the HBL results.

### Implications for clinical practice

While APCs have not yet been established as the gold standard for socket preservation, their use—particularly in conjunction with bone grafts—has been suggested in the literature. From this review, the decision to employ APCs should be informed by the clinician’s knowledge and experience, as their efficacy is suggested but the certainty in the evidence is not high; further clinical trials are necessary to enhance this certainty. Regarding safety, no complications or adverse events related to the use of APCs have been reported in the literature to date. However, their use is not without drawbacks, including the additional costs of specialised equipment such as centrifuges, increased chair time required for blood withdrawal, and the need for additional training to ensure the optimal handling and application of APCs

### Limitations

The inherent heterogeneity among the selected studies introduces potential inconsistencies in the findings. Furthermore, a significant limitation is the moderate-to-high risk of bias present in 2 out of 5 studies (40% of studies), alongside a ‘Low’ GRADE score for evidence quality assessment, which reduces the overall validity and reliability of the synthesized results.

Additionally, the inclusion criteria restricted the selection to studies published exclusively in English, potentially excluding relevant articles that could either support or challenge the conclusions of this review. Moreover, the time range of the search, spanning from January 2014 to August 2024, may not include all the developments in the field. Finally, the present meta-analysis was not registered online. This could result in unplanned duplication and prevents the verification that the methods were carried out as planned.

### Recommendations for future trials

Future clinical trials should prioritise measuring the benefits associated with newer generations of APCs, such as PRF, A-PRF, A-PRF+, i-PRF, or CGF with bone grafts in reducing VBL and HBL as a mean of socket preservation. We recommend an interval follow-up period of 4–6 months for measuring changes in bone, which aligns with the socket healing time required for the delayed implant placement protocol. It is essential for the participants to be randomized with allocation concealment maintained until the time of intervention, while outcome assessors should remain blinded to the intervention allocation to minimise bias. Additionally, it would be advantageous to investigate which baseline characteristics may influence the extent of benefits derived from this innovative socket preservation technique. Factors such as patient age, sex, extraction site, medical history, smoking status, diabetes, oral hygiene and history of periodontal disease should be examined. This knowledge would provide practitioners with valuable insights for optimising case selection when implementing APCs.

## Conclusion

This review identifies the benefits of combining APCs with bone grafts for socket preservation, particularly in reducing VBL. While significant effect sizes were observed in certain studies, the variability in results across different studies shows heterogeneity in this area of research. The meta-analysis indicates a medium-to-large effect on VBL, and a small-to-large effect on HBL outcomes, where only one study demonstrated a statistically significant effect. It is important to note that, while statistical significance was observed for large effect sizes in the use of APCs combined with bone grafts compared to bone grafts alone, the GRADE assessment classifies the overall quality of evidence as low for horizontal bone loss and moderate for vertical bone loss.

The limited sample size of five identified studies evaluating 126 extraction sites is inadequate to confirm the benefit for clinical application of combining APCs with bone grafts. Therefore, while the current evidence suggests that APCs may enhance the effectiveness of bone grafts, further clinical trials are needed to add certainty to their efficacy. Well-designed RCTs, preferably multi-centre studies with larger sample sizes, may generate robust conclusions supporting evidence-based practice in this context. The recommendations for future research, including the investigation of newer generations of APCs and their impact on various patient characteristics, will contribute to a more nuanced understanding of their role in socket preservation.

As further evidence is identified, clinicians are encouraged to remain informed and apply their knowledge and experience when considering the use of APCs in practice.

## Supplementary information

Prisma Checklist

Supplement 1a 1b

## Data Availability

The data that support the findings of this study are available from the corresponding author upon reasonable request.
