# Supplementary material for: Vertical and horizontal bone loss following alveolar socket preservation using bone grafts and autologous platelet concentrates vs bone grafts alone: a systematic review and meta-analysis
Source: BDJ Open. 2025 Feb 6;11:11. doi: 10.1038/s41405-025-00306-y (PMC11802899; doi:10.1038/s41405-025-00306-y)
Supplement: Supplementary file 2 — Supplement 1a 1b [file 41405_2025_306_MOESM2_ESM.docx]

**Supplement 1a**. Search key words.

A table showing the search strategy employed in PubMed, Scopus, Embase and Google Scholar to identify the articles for this review.

| **PubMed search** | **Scopus** | **Embase** | **Google Scholar search** |
| --- | --- | --- | --- |
| (platelet*) AND (preserv* OR ridge* OR socket*) | TITLE-ABS-KEY ((platelet*) AND (preservation* OR ridge* OR socket*)) | (platelet*) AND (preservation* OR ridge* OR socket*) | (platelet OR platelets) (socket OR sockets OR ridge OR preserve OR preservation) |

**Supplement 1b**. Inclusion and exclusion criteria.

A table showing the inclusion and exclusion criteria used during the articles selection process which led to the final inclusion of the studies in this review.

| **Inclusion criteria** | **Exclusion criteria** |
| --- | --- |
| - Hospital or clinical setting - Adult population (18-75 years old) - Randomized controlled trials - Studies utilising APCs in extraction sockets - Studies reporting radiographic vertical bone loss and horizontal bone loss. - Studies comparing at APCs+bone graft vg bone graft-alone - The investigation period is between 01^st^ of January 2014 and 24^th^ of August 2024 - No restrictions on type of APCs - No restriction on study sample size - No restrictions on geographic location | - Studies without a graft-only group - Studies not reporting radiographic bone loss - Paediatric or special needs population - Restorative or endodontic treatment - Non-dental procedures - Animal studies, books, reviews, case reports, editorials, letters, commentaries, or conference abstracts - Duplicate studies - Non-English articles |
